# Effect of implant neck design on peri-implant tissue conditions: a narrative review

**DOI:** 10.1186/s40729-026-00683-5

**Published:** 2026-04-15

**Authors:** Ziqi Xie, Sara Reda Sammour, Toru Ogawa, Nobuhiro Yoda

**Affiliations:** 1 Division of Advanced Prosthetic Dentistry, Tohoku University Graduate School of Dentistry, Sendai, Japan; 2 Division of Comprehensive Dentistry, Tohoku University Graduate School of Dentistry, Sendai, Japan

**Keywords:** Dental implants, Osseointegration, Implant neck design, Peri-implant tissue, Marginal bone loss

## Abstract

**Purpose:**

The neck design of dental implants, which is crucial for the long-term health of the surrounding periodontal tissue, affects both the marginal bone preservation and soft tissue integration. This narrative review investigates various approaches for implant neck design, including macroscopic contour profiles (straight, divergent, convergent, scalloped, sloped, and triangular), surface texture modifications (microthreaded, roughened, and machined collars), and platform switching.

**Methods:**

A narrative literature search was conducted in PubMed, Scopus, and Web of Science up to July 2025. Studies examining implant neck geometry, surface characteristics, or their mechanical and biological relevance were included. Evidence from finite element analyses, preclinical studies, clinical investigations, and systematic reviews or meta-analyses was qualitatively synthesized for comparing the implant neck designs.

**Results:**

Finite-element analyses and clinical trials have indicated that divergent and convergent necks exhibit better biomechanics than traditional straight-neck designs. Sloped and triangular designs can adapt to the ridge shape, thus improving soft tissue appearance. Microthreaded and roughened collars may optimize stress distribution and promote osseointegration; however, certain concerns, such as the risk of bacterial colonization, exist. Platform switching has demonstrated consistent benefits in reducing crestal bone loss, particularly in the esthetic zones and immediate placement protocols. However, the overall evidence remains heterogeneous across studies.

**Conclusions:**

This review highlights the need to shift from a design-centric to an indication-oriented strategy. Instead of relying solely on the manufacturer’s claims, neck designs should be altered based on specific factors such as tissue morphology, esthetic considerations, hygiene accessibility, and load protocols.

## Background

Dental implants have emerged as a widely accepted and reliable treatment modality for partially or completely edentulous patients [1–3]. Although recent advancements in the design and surgical protocols have improved the implant survival rates [4, 5], clinical success is no longer assessed solely by the achievement of osseointegration or implant survival, but also by the preservation of marginal bone and peri-implant soft tissues, both of which are critical for sustained function and esthetic outcomes [6].

Early peri-implant bone loss is commonly observed as a consequence of physiological bone remodeling during the initial phase of healing. Among various factors influencing peri-implant bone preservation, the design of the implant neck has emerged as a critical determinant [7]. This region, located at the interface between the implant and the crestal bone, is highly susceptible to biomechanical stresses [8]. Once exposed to the oral cavity, the dental implant surface serves as a platform for microbial adhesion and biofilm accumulation [9], further contributing to marginal bone loss (MBL) around the implant neck. Therefore, the implant neck is a critical region directly affecting biomechanical stability and peri-implant tissue health.

Although the critical role of the implant neck in peri-implant tissue stability is well-recognized, the optimal design configuration remains uncertain. A wide range of neck design strategies has been introduced to enhance biomechanical performance, preserve marginal bone, and promote soft tissue integration. These include variations in the macroscopic geometry, surface characteristics, and platform-switching concepts, all of which have been the subject of extensive biomechanical and clinical investigations.

Although several studies have investigated the effects of individual implant neck design features, the findings remain heterogeneous across different design dimensions. Conflicting results have been reported concerning neck contours [10, 11] and surface textures [12]. Given the wide variety of implant systems currently available, clinicians are frequently confronted with complex choices regarding neck design; however, no unified framework exists to guide decision-making.

This narrative review provides a comprehensive overview of current implant neck design approaches, including macroscopic geometry, neck surface properties, and platform-switching designs. We investigated the biomechanical and biological concepts underlying each strategy using finite element analyses (FEAs), preclinical investigations, and clinical data.

Rather than aiming to rank individual designs, we highlight the contexts in which specific configurations may offer advantages for marginal bone preservation and peri-implant soft tissue stability, and offer a comprehensive framework for clinical selection. A comparative summary of the key design features and clinical considerations is presented in Table 1 to aid interpretation in the following sections.

**Table 1:** A comparative summary of major implant neck design characteristics and associated clinical implications

Design domain	Feature type	Design classification	Design rationale	Biomechanical findings	Clinical evidence	Indications
Macroscopic Geometry Design	Neck Contour Profile Design	Regular (Straight)	Features a platform continuous with the implant body; simple design with long-standing clinical use	Highest stress concentrations in the cortical and cancellous bone [13]	No significant survival or MBL differences found between straight, divergent, and convergent designs after 1 year [14]	Widely used; suitable for general cases, but may be biomechanically less favorable under lateral loading
		Divergent (Back-tapered)	Broader coronal platform aimed at reducing the shear force and enhancing stress distribution, especially under lateral load	Produced lowest von Mises and principal stress values among profiles in the FEAs [15, 16]; however, it showed favorable distribution [17]	Less MBL and PPD than convergent design in the posterior regions [18] Not superior in the survival rate [14]	Preferred in the posterior regions or cases with high occlusal load requiring mechanical stability
		Convergent (Tapered)	Narrowing coronal profile to enhance soft tissue support; creates subcritical contours beneficial to tissue thickness	Reduced stress in the tapered designs [13] Favorable stress under vertical load; however, the evidence remains mixed [17]	Less bone loss than divergent design [19]	Suitable for esthetic zones with thin buccal bone and soft tissue concerns; supports soft tissue healing and PES outcomes
	Anatomically Shaped Neck Design	Scalloped	Mimics the natural CEJ and alveolar crest anatomy to enhance soft tissue contour and esthetics	Not specifically emphasized	More bone resorption [10]; no esthetic or patient satisfaction benefit [11]; no additional benefits concerning MBL or prosthetic complications when compared to flat platform designs [20]	Initially proposed for the anterior esthetic zones, but current evidence does not support routine use
		Sloped	Follows the buccal–lingual ridge slope, aiming to reduce grafting needs and preserve vertical bone anatomy	Reduced von Mises stress and better ridge adaptation [21]	Stable marginal bone and soft tissue [22, 23]; a progressive increase in the width of the keratinized peri‐implant mucosa during follow-up [22, 24]; minimal marginal bone alteration in the immediate protocols [25]	Suitable in regions of sloped ridges, fresh extraction sockets, and esthetic areas with thin buccal bone
	Novel Neck Shape Design	Triangular Configuration	Flat surfaces reduce cortical bone contact to minimize crestal stress; gaps promote bone infill	Not specifically emphasized	Improved PES and stable hard and soft tissues [26, 27]; Comparable survival and success rates (97.5% and 96.7%, respectively) [28]	Immediate placement protocol with a high esthetic demand, such as the anterior maxilla [29]
Implant neck surface property Design	Neck surface Design	Microthreaded	Designed to optimize stress distribution and reduce compressive stress peaks at the marginal bone interface, thereby minimizing marginal bone loss	Lower stress in cortical bone compared to smooth designs [30]; Protective effect may diminish over time due to marginal bone resorption [31]	Long-term high survival rate (97.9%) and stable peri-implant conditions [32]; Reduced MBL [33]; Higher primary stability than machined collars [34]	Preferred for early osseointegration and primary stability; especially suitable for immediate loading protocols
		Roughened	Moderate roughness improves osseointegration by enhancing bone–implant contact	Not assessed independently	Reduced peri-implant MBL and probing depths compared to machined collars [35]	Enhances bone–implant contact and is beneficial for achieving strong osseointegration in the early healing phase
		Machined (Smooth)	Designed to reduce bacterial adhesion and enhance peri-implant hygiene by limiting microbial colonization	More stress in the cortical bone compared to microthreaded design [30]	Less CBL in early healing compared to the microthreaded designs [34]; supracrestal placement associated with better outcomes [36, 37]	Suitable for maintaining soft tissue health and hygiene, particularly when placed supracrestally Not recommended for subcrestal placement
Connection Design	Implant–Abutment Interface Design	Platform Switching	A horizontal mismatch between the implant platform and abutment to relocate the microgap inward, minimizing crestal bone loss	Reduced crestal bone stress than platform matched [38, 39]	Reduced MBL compared with platform matched [40, 41]; reduced probing depths, improved PES compared to platform-matched designs [41]; and minimal marginal bone loss and favorable soft tissue outcomes in immediate placement cases [42, 43]	Esthetic zones, immediate loading or placement cases, and sites with thin crestal bone. Use should be case-specific based on the tissue morphology and prosthetic needs

MBL: marginal bone loss; PES: pink esthetic score; PPD: periodontal probing depth; CBL: crestal bone loss, CEJ: cementoenamel junction

## Methods

A narrative, non-systematic search was conducted in PubMed, Scopus, and Web of science using combinations of the following terms: “implant neck design,” “implant neck,” “implant collar,” “neck geometry,” “macrogeometry,” “straight neck,” “convergent neck,” “divergent neck,” “scalloped neck,” “sloped neck,” “triangular neck,” “microthreads,” “machined surface,” “roughened collar,” and “platform switching.” No temporal restrictions were applied, and the search was updated through July 2025. Additional articles were identified through citation screening.

Studies were included if they described the morphological characteristics of the implant neck or collar region or investigated their mechanical or biological relevance, or provided synthesized evidence such as systematic reviews or meta-analyses. Owing to methodological diversity across studies, comparative evaluation was structured qualitatively rather than through a single standardized metric. Evidence was organized into three consistent domains—biomechanical analyses, preclinical tissue-level findings, and clinical outcomes—to enable coherent comparison across different neck designs.

## Macroscopic geometry design

### Neck contour profile design

The macroscopic contour of the implant neck plays a crucial role in osseointegration and distribution of mechanical stress to the surrounding crestal bone. Based on a comparison with the implant body diameter, implant neck contour profiles are commonly categorized into regular (straight), divergent (back-tapered), and convergent (tapered) necks.

**Fig. 1:**
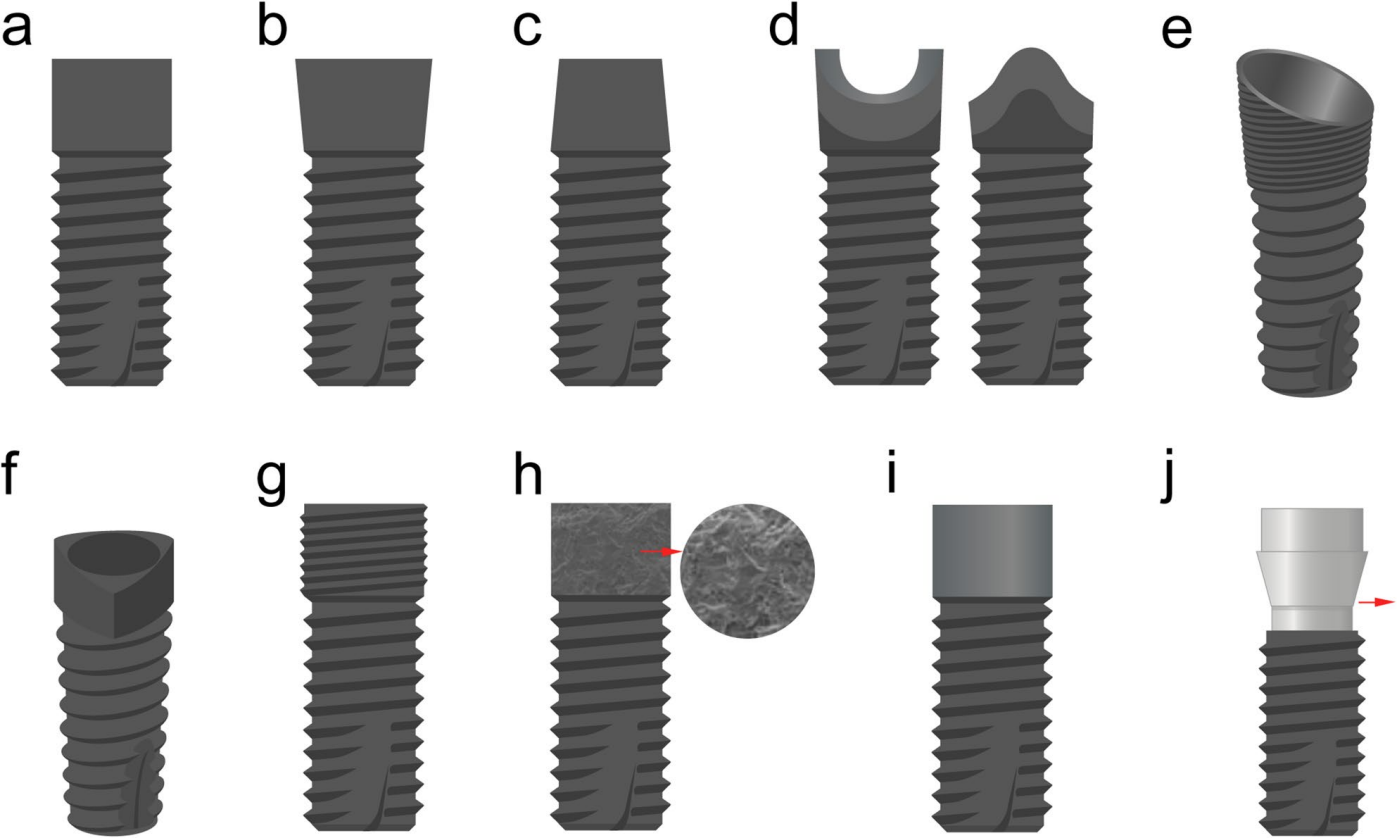
Schematic representation of three neck designs: Dental implants are available in both straight and tapered configurations; however, this figure illustrates only the classification of the neck region, with all implants shown being bone-level designs. **a** Regular (straight) neck; **b** Divergent (back-tapered) neck; **c** Convergent (tapered) neck; **d** scalloped neck design Left: mesiodistal view; Right: buccolingual view. **e** sloped neck design; **f** triangular neck design; **g** Microthread neck design; **h** Roughened surface (mimicking scanning electron microscopy topography); **i** Machined smooth neck design; **j** platform switching design Red arrow indicates the abutment

Regular necks feature a platform that is continuous with the implant body, whereas divergent necks are characterized by a broader platform that is believed to improve the stress distribution by reducing shear forces, particularly under lateral loading conditions. Conversely, convergent necks exhibit a gradually narrowing coronal profile, which may cause stress concentration in the adjacent compact bone [15]. A schematic representation of the three neck designs is shown in Fig. 1a–c.

These modifications have predominantly been investigated and applied in bone-level implant systems. FEA studies investigating the biomechanical implications of implant neck geometry have reported inconsistent outcomes. Bozkaya et al. [17] concluded that convergent collars exhibited superior stress distribution than divergent designs under vertical loading conditions; however, the potential confounding influence of thread geometry was not accounted for. In contrast, Shen et al. [15] evaluated straight, convergent, and divergent profiles and identified the divergent neck as producing the lowest von Mises and principal stress values. This finding was further supported by Panmei et al. [16], whose comparison of divergent and convergent configurations similarly favored a divergent design. Additionally, Ishak et al. [13] reported that straight-neck implants induced the highest stress concentrations in both cortical and cancellous bone, whereas 15° and 30° tapered (convergent) necks resulted in significantly reduced stress levels. Collectively, although convergent and divergent designs have demonstrated relative advantages over straight-neck configurations, the literature remains divided regarding their comparative efficacy.

Interestingly, although convergent necks may lead to localized stress concentration in the adjacent compact bone, they have demonstrated favorable outcomes in soft tissue management. The tapered coronal profile reduces the surface area and provides more regenerative space, facilitating soft tissue thickening and stability. This design concept aligns with contemporary restorative guidelines that advocate the use of subcritical concave emergence profiles to promote peri-implant soft tissue health [44].

Clinical research has provided conflicting findings regarding the impact of the implant neck design on clinical outcomes. Moreover, the superiority of one neck shape over another remains unclear. A systematic review by Messias et al. [14] revealed no statistically significant differences in implant survival or MBL among straight-, divergent-, and convergent-neck designs after 1 year of follow-up. In contrast, a 2-year follow-up study comparing rough-surfaced divergent and convergent neck implants in the molar–premolar region found that divergent neck designs exhibited less MBL and shallower probing depths, with comparable survival rates [18]. Agustín-Panadero et al. [19] found that implants with a convergent transmucosal neck exhibited significantly reduced peri-implant bone loss compared than those with a divergent transmucosal neck. The clinical evidence supports these biomechanical findings.

However, the implant neck contours have rarely been evaluated in isolation. Modern implant systems incorporate multiple concurrent design modifications, making delineating the specific biomechanical and biological contributions of a single collar profile challenging. The Prama® implant system (Sweden & Martina SpA, Due Carrare, Padua, Italy) exemplifies an intramucosal, convergent-neck design integrating multiple features aimed at enhancing soft tissue outcomes and long-term esthetics. This system incorporates a convergent transmucosal collar and microtextured surface (Ultrathin Threaded Microsurface, UTM) to promote connective tissue attachment [45]; furthermore, the elimination of an abutment-level finishing line enables prosthetic-driven soft tissue shaping [46].A systematic review reported that the Prama implant system achieved a satisfactory survival rate, with stable or improved hard tissue parameters (such as marginal bone level) and soft tissue parameters (such as the pink esthetic score [PES]) throughout the follow-up period [47].

While convergent necks may enhance soft-tissue stability in esthetic zones with thin buccal bones, the use of divergent profiles be more suitable for posterior regions warranting greater mechanical stability. However, these benefits require further confirmation through high-quality prospective studies.

### Anatomically shaped neck design

#### Scalloped neck design

Scalloped neck implant designs were originally introduced to mimic the natural cemento-enamel junction and follow the three-dimensional anatomy of the anterior alveolar crest. This design is intended to improve the esthetic outcomes, particularly in the anterior region [48]. A representative illustration of the scalloped neck implant is shown in Fig. 1d.

However, accumulating clinical evidence does not support these hypotheses. A systematic review demonstrated that scalloped implants exhibited significantly greater amount of peri-implant bone resorption than those with a flat implant–abutment shape [10]. Another systematic review concluded that modifications in the shoulder design of scalloped, sloped, and one-piece implants did not contribute to superior outcomes in terms of marginal bone maintenance, esthetics, or patient satisfaction [11]

These findings were further supported by a 4-year follow-up clinical study that reported that the use of scalloped cemented implants (Bone System s. r. l., Scalloped, Milan, Italy) did not offer additional benefits in terms of MBL or prosthetic complications when compared to flat platform designs [20]. To better understand the discrepancy between the anticipated advantages and actual clinical outcomes of scalloped implants, we have summarized the possible reasons reported in the literature in Table 2.

**Table 2:** Summary of the potential reasons for the mismatch between the proposed advantages and clinical outcomes of scalloped implants

Potential factors	Explanation	References
Increased marginal bone loss	Scalloped implants showed more bone resorption, potentially leading to papilla loss	[48–50]
Anatomical complexity	Papilla development is multifactorial Interdental papilla formation depends on various anatomical and biological factors—including bone thickness, tooth morphology, and soft tissue architecture—that cannot be reliably controlled by the implant shoulder design alone	[48, 49]
Alveolar bone variability	Highly variable alveolar contour limits the adaptability of uniform scalloped designs	[48]
Large implant diameter	Greater diameter (5.3 mm) may lead to excessive bone removal and vascular disruption	[20]
	Scalloped implants with larger diameter (4.31 mm) compared to flat implants (3.54 mm), often placed at lateral incisor sites, may result in an interimplant distance of less than 3 mm, leading to overlapping bone resorption areas and increased horizontal and vertical bone crest loss	[50]
Index insensitivity	Bone loss may be too minor to be detected by esthetic indices	[51]
Adjacent tooth influence	The bone level next to the adjacent teeth is highly related to at least the future level of the papillae. Possibly, the periodontium also acts on other esthetically related aspects as the level of the facial mucosa	[51]
Preoperative mucosal level	The level of the mucosa before implant placement was more relevant to the future level of the peri-implant mucosa than the amount of bone loss around the implant neck	[51]

This evidence suggests that scalloped platforms may provide limited benefits. The lack of persistent clinical advantage may account for the declining academic interest in this design in current publications.

#### Sloped neck design

Sloped neck implants are designed to follow the natural buccal–lingual ridge anatomy, allowing the preservation of vertical discrepancies between marginal bone levels. This configuration improves the implant adaptation to sloped ridges and may reduce the need for additional bone augmentation procedures [52, 53]. A representative illustration of a sloped neck implant is shown in Fig. 1e.

Their biomechanical advantages were demonstrated using FEAs. For instance, an FEA study demonstrated that implants with a sloped marginal configuration (Medentika, Straumann Group, Calw, Germany) could be placed without additional bone grafting, thereby simplifying the surgical procedure. This design offers a non-invasive and cost-effective alternative to conventional flat-neck implants, which typically require advanced surgical interventions during placement [54]. Furthermore, another study found that 30° tilted sloped-neck implants exhibited lower von Mises stress values (158.489 MPa) than conventional flat-neck designs (177.208 MPa) in the all-on-four protocol, indicating better load distribution and reduced risk of bone-related complications [21].

OsseoSpeed™ Profile TX is an example of sloped configuration implant system. It features a fluoride-modified surface (OsseoSpeed), microthreads at the neck region, a conical Seal Design, and a connective contour aimed at supporting the peri-implant soft tissue as stated in the manufacturer’s documentation (Dentsply Sirona, 2023).

Sloped configurated implants (OsseoSpeed™ Profile TX) have been associated with high survival rates, and their shoulder design contributes to enhanced support of the peri-implant hard and soft tissues [22, 23]. Furthermore, longitudinal studies have reported a progressive increase in the width of the keratinized peri-implant mucosa during follow-up [22, 24].

Multiple design components of the OsseoSpeed™ Profile TX system likely contribute to the peri-implant tissue response observed in clinical studies. A fluoride-modified surface increases bone-to-implant contact (BIC) during the early stages of healing [55, 56]; the microthreads at the collar reduce crestal stress, promoting marginal bone stability [30, 33]. Moreover, the conical implant–abutment connection improves mechanical sealing [57]. These elements, in conjunction with the sloped configuration, may account for the favorable hard and soft tissue outcomes reported.

OsseoSpeed™ Profile TX implants have been successfully applied in immediate temporization protocols. Clinical and radiographic findings suggest that, sloped implants inserted into extraction sockets and provisionalized immediately, even in cases with facial bone wall deficiencies, demonstrate favorable preservation of the marginal bone and maintenance of peri-implant soft tissue esthetics [58]. Furthermore, a 5-year prospective study confirmed that the OsseoSpeed TX Profile implants placed in fresh extraction sites with immediate temporization exhibited minimal marginal bone alterations and achieved long-term outcomes comparable to those of conventional regular neck implants [25].

Thus, sloped-neck configurations allow better adaptation to anatomical variations and have demonstrated reduced stress distribution and satisfactory clinical performance, particularly in cases of immediate temporization.

#### Novel neck shape design: triangular configuration

Recently, an implant design featuring a triangular neck configuration (V3, MIS; Bar-Lev Industrial Zone), which differs from the traditional circular neck design, has been introduced. A schematic of the triangular neck design is shown in Fig. 1f. When the implant is placed at the crestal level, the flat surfaces of the triangular neck remain largely disengaged from direct contact with the surrounding cortical bone [59, 60]. Consequently, only 27–29% of the implant neck perimeter contacts the adjacent supporting bone [61]. This design concept was intended to reduce the mechanical stresses and strains transmitted to the crestal bone during implant seating, thereby potentially preserving the marginal bone levels.

A human histological study reported that triangular-neck implants achieved a mean BIC ranging from approximately 64–69% across the buccolingual, mesiodistal, and circumferential planes, suggesting that triangular neck implant designs allow the creation of a gap between the osteotomy site and implant surface, resulting in subsequent bone apposition and osseointegration [62].

Preclinical evidence for the efficacy of triangular neck implants remains inconclusive. One animal study reported that triangular implants demonstrated osseointegration rates, buccal bone volume, and soft tissue contours similar to those of conventional circular neck implants. The only exception was a significantly greater buccal crestal bone width observed in the triangular group, while no significant differences were observed in the soft tissue dimensions or the location of the first BIC [60]. Another study suggested that the triangular neck implant design exhibited a greater thickness of the peri-implant tissue than conventional designs [26].

Clinically, one retrospective study demonstrated a significant improvement in the PES as well as excellent hard and soft tissue preservation after 1 year of function, thus allowing immediate placement of triangular-shaped neck implants in areas with a high esthetic demand, such as the anterior maxilla [29]. A randomized controlled trial (RCT) further suggested that triangular designs may result in less crestal bone loss than conventional implants while demonstrating comparable outcomes in both hard (Clinical Buccal Bone Thickness, Implant Stability Quotient and soft tissue parameters (Pocket Probing Depth [PPD], Plaque Index, and Gingival Index) [27]. A 1-Year prospective cohort study reported comparable survival and success rates for triangular neck implants and indicated that factors such as smoking may increase the risk of implant failure, highlighting the need for careful patient selection [28]. A 5-year RCT found no significant differences between triangular and round-neck implants in terms of peri-implant bone changes, PES, or patient-reported outcomes [63].

While certain studies have demonstrated favorable outcomes for triangular neck implants in terms of peri-implant tissue preservation and esthetics, the overall evidence is inconclusive due to wide variations in the sample size, follow-up duration, study design, confounder control, implant site location, and healing conditions. Thus, whether triangular neck designs provide any advantages over traditional circular designs remains uncertain, highlighting the need for further high-quality longitudinal research.

## Implant neck surface property designs

Beyond the macroscopic contours, the structural and biological features of the implant neck, particularly its interface with the peri-implant bone and soft tissue, play crucial roles in long-term stability. Among them, microthreaded, roughened, and machined (smooth) collars have been widely studied for their mechanical and biological properties. A schematic representation of the implant neck surface design is shown in Fig. 1g–i.

### Microthreaded collars design

Microthreads are retention elements provided in the form of small threads located at the implant neck. They are designed to optimize the stress distribution at the marginal bone interface and reduce compressive stress peaks, thereby minimizing the MBL [64]; hence, they are commonly used in bone-level implants.

FEAs have demonstrated that microthreaded necks reduce stress concentrations in the adjacent cortical bone and promote favorable stress distribution compared to machined designs [30]. However, the protective biomechanical effect may diminish over time owing to progressive marginal bone resorption [31].

Clinical evidence supports the use of micro-threaded collars. Although one meta-analysis found that microthreads reduced MBL, the number of RCTs included was limited [33].

To optimize their performance, efforts have been made to optimize the microthread geometry for enhanced biomechanical outcomes. An animal study with numerical analysis indicated that V-shaped threads provide superior stress distribution and osseointegration than power-shaped threads; however, the microthreads extend along the entire implant body rather than being confined to the neck [65]. Another analysis found that coarse microthreads generated less shear stress than fine ones, and that a 3 mm microthreaded section yielded better biomechanical outcomes than 1 mm or 2 mm [66]. A more recent FEA compared five microthread shapes (straight, square, V-shaped, buttress, and reverse buttress) and found that the square-shaped threads produced the lowest von Mises stress along the implant body [67].

Although such geometric refinements are still being evaluated in preclinical settings, clinical evidence has demonstrated that microthreaded collars yield favorable outcomes. A long-term retrospective analysis reported a 97.9% cumulative survival rate for microthreaded implants, with stable peri-implant conditions [32].

### Roughened collars design

Human histological evidence indicates that roughened surfaces yield higher BIC%, greater intra-thread bone area, and increased bone density, thereby enhancing early bone healing under unloaded condition [68].However, the biological advantages of surface roughness are counterbalanced by potential risks. Roughened titanium surfaces can facilitate bacterial adhesion and accelerate biofilm maturation by providing larger microbial retention sites [69]. Further, recent investigations focusing specifically on the coronal/neck region demonstrate that surfaces with surface roughness (Sa) values > 1 μm promote significantly higher early adhesion of *Streptococcus oralis*, an important early colonizer in peri-implant biofilms, suggesting that excessively rough neck surfaces may increase the likelihood of soft-tissue inflammation [70].

Roughness plays a crucial role in the initial stability and long-term success of implants. A systematic review reported that laser microtextured (roughened) collars significantly reduced peri-implant MBL and probing depths compared to machined collars; however, no significant difference was observed in the implant failure rates [35], which may be attributed to enhanced BIC resulting from moderate surface roughness. However, the potential benefits of increased roughness should be balanced against the increased risk of bacterial colonization and subsequent peri-implant inflammation [71].

Owing to their respective mechanical and biological advantages, microthreads and surface roughening can be combined to achieve synergistic effects. A systematic review confirmed that under functional loading, a roughened microthreaded design for the implant neck could significantly lower the MBL. Furthermore, PPD and MBL were much lower around laser microtextured neck implants than around machined necks or microthreaded implants [12].

### Machined collars design

Although roughened and microthreaded collars offer biomechanical advantages, such as enhanced osseointegration, their surface characteristics, especially increased roughness, may also promote bacterial adhesion and plaque accumulation [72].

Machined collars have been developed to enhance peri-implant hygiene and reduce the risk of peri-implant diseases by limiting microbial colonization [73].

However, direct comparisons between collar types remain complex owing to potential confounding factors such as implant placement depth, which may critically affect the interpretation of MBL outcomes. A recent study included in the systematic review by Huraib et al. [12] reported no significant differences between machined and roughened collars in terms of the MBL, peri-implantitis rate, implant survival, or hard and soft tissue integration [74]. Additionally, clinical investigations have suggested that machined collars offer specific advantages under certain conditions. For instance, a split-mouth comparative study found that machined collars exhibited significantly less crestal bone loss during the initial 3-month healing period than microthreaded collars, likely owing to lower plaque accumulation and reduced insertion stress, which resulted in significantly higher primary implant stability, potentially making them more suitable for immediate loading protocols [34].

Vertical placement is a critical factor affecting the performance of machined collars. A systematic review indicated that supracrestal positioning (placing the collar above the alveolar crest) yields more favorable outcomes in both one- and two-piece implant systems [36]. This trend was further supported by a recent study reporting that supracrestal placement was associated with stable marginal bone levels, whereas subcrestal positioning resulted in significantly greater bone resorption over time [37].

The pronounced bone remodeling often observed with subcrestally placed machined collars may be attributed to their limited osteoconductivity, as their smooth surfaces fail to adequately stimulate bone apposition [75]. A systematic review concluded that subcrustal placement should be avoided in machined neck implants owing to the increased risk of bone loss [76]. Although machined collars were associated with slightly improved probing depths and marginal bone levels, these differences were not statistically significant. In contrast, rough-surface implants exhibited lower failure rates [77]. These findings suggest that machined collars offer soft tissue benefits without compromising the bone stability in supracrestal positions.

Therefore, a microthreaded design may be preferred in cases where early osseointegration is desired, such as in an immediate loading protocol, owing to its biomechanical advantages. Conversely, a machined collar at the soft-tissue interface may be considered more suitable for cases prioritizing long-term maintenance of peri-implant hard- and soft-tissue health and favorable esthetic outcomes.

## Platform switching at the implant neck

Although often classified under implant–abutment connection strategies, platform switching directly influences the stress distribution and biological response in the implant neck region. The concept of platform switching emerged in 1991 when narrower abutments were placed on wide-diameter implants, resulting in reduced MBL [78]. It involves using a prosthetic abutment with a smaller diameter than the implant platform and shifting the implant–abutment junction (IAJ) medially to minimize crestal bone loss [79]. A schematic representation of the platform switching at the implant neck is shown in Fig. 1j.

The biological rationale of platform switching is grounded in the establishment of peri-implant biological width. Berglundh et al. proposed that biological width existed around implants [80], conceptualized as a dimension of approximately 3–4 mm from the top of the peri-implant mucosa to the first bone-to-implant contact, acting as a protective barrier for peri-implant tissues [81]. Platform switching displaces the IAJ medially, thereby increasing the horizontal biological width and positioning the microgap further from the crestal bone [82]. Because inflammatory cell infiltrate typically accumulates around the IAJ [83], this medial repositioning redirects the infiltrate toward the central axis of the implant rather than toward the outer crestal bone, reducing microgap-associated inflammatory stimulation. At the same time, the lateral connective-tissue compartment becomes thicker and more densely vascularized. Collectively, these adaptations allow the biological width to re-establish coronally rather than migrate apically, thereby minimizing early crestal bone remodeling [84, 85].

Several FEA studies have supported the biomechanical rationale of platform switching, which consistently revealed that platform-switched implants exert less stress on the crestal bone than platform-matched implants. Building on this foundation, individual studies provided additional insights. One study demonstrated that platform switching resulted in a measurable but minimal effect on the von Mises stress in the crestal region of the cortical bone, suggesting that its mechanical benefits may be limited [38]. Another study reported that platform switching shifted the stress concentration from compact to cancellous bone under oblique loading [39].

Favorable clinical outcomes are obtained by platform switching. A systematic review and meta-analysis revealed that platform-switching implants exhibited a 0.22 mm reduction in crestal bone loss than conventional designs [40]. A recent long-term retrospective study also supported this finding. A 10-year cohort study in the anterior maxilla demonstrated significantly lower MBL, reduced probing depths, and improved PES for platform-switching implants compared with platform-matched designs [41]. Platform-switching implants have shown promising results in immediate loading protocols. A prospective study evaluating immediately loaded Nobel Biocare implants with a built-in platform-switch design in the maxillary esthetic zone reported favorable clinical outcomes. Over a 36-month follow-up period, the implants exhibited minimal MBL (≤ 0.2 mm annually), stable peri-implant soft tissue conditions, a gingival thickness of 3.47 ± 0.34 mm and a keratinized tissue width of 2.46 ± 0.39 mm, and achieved successful osseointegration with an acceptable esthetic appearance [42]. A systematic review also supported this clinical applicability, concluding that platform switching is particularly advantageous in immediate placement protocols; however, the evidence remains limited by clinical heterogeneity and potential confounders [43].

In summary, platform switching is an effective design for minimizing early MBL, particularly in esthetic zones or for cases of immediate implant placement. However, its application should be case-specific, including bone morphology, soft-tissue thickness, and prosthetic specifications, rather than being used as a universal standard. This is particularly relevant when limited bone width precludes the placement of larger implants or when smaller-diameter abutments may affect the emergence profile in the anterior regions [82].

## Biomechanical basis of implant neck design

The remodeling of bone at the implant neck is strongly influenced by the local mechanical environment. Variations in the neck geometry alter how functional loads are transferred, resulting in different strain distributions at the bone–implant interface. Wolff [86] first proposed that bone structure adapts to habitual loading, and Roesler [87] later refined this concept by articulating the structure–stress relationship. Consistent with these principles, both observational data and finite element analyses have demonstrated that the crestal cortical bone—where local stresses are highest—is particularly vulnerable to early bone resorption [88]. Although the included studies differed in implant geometry and in the specific stress variables analyzed, their finite element comparisons consistently assessed stress patterns within the crestal bone surrounding the implant neck. Therefore, implant neck design should account for its mechanical influence on this region.

## Conclusions

To ensure consistent evaluation across diverse implant neck configurations, evidence in this review was organized into three domains—biomechanical analyses, preclinical tissue-level findings, and clinical outcomes—to enable coherent comparison of different neck designs. Despite the diversity of implant neck designs, ranging from macro-contour modifications to surface improvements and platform-switching strategies, current evidence remains inconsistent. Some clinical studies assessed implant systems as a whole, making it challenging to isolate the effects of neck-specific features. Although numerous theories, such as platform switching, exhibit encouraging developments, the long-term effectiveness of novel shapes, such as triangular or scalloped necks, is unclear owing to limited follow-up and diverse outcome evaluations.

Future studies require multifactorial designs that consider neck geometry in conjunction with surface texture, placement level, soft- and hard-tissue conditions, and prosthetic emergence profiles. All neck designs considered in this review are bone-level, which provided the framework for the present analysis. Creating standard definitions and reporting criteria for neck design would enhance consistency across studies.

To facilitate an indication-driven interpretation of the evidence, Table 3 organizes the clinical scenarios reviewed in this study alongside the implant neck configurations reported in the literature for each clinical indication. Clinically, this highlights the need to shift from a design-centric approach to an indication-driven approach. Neck designs should be customized based on particular conditions, such as the tissue morphology, esthetic demands, oral hygiene accessibility, and loading protocols, rather than relying solely on the manufacturer claims.

**Table 3:** Clinical indication–driven selection of implant neck designs

Clinical indication	Recommended neck design(s)
Esthetically critical anterior region, especially with thin buccal bone	Convergent neck/ Triangular neck/ Platform switching
Immediate placement / immediate temporization (e.g., thin facial plate, extraction sockets)	Sloped neck/ Triangular neck/ Platform switching
Posterior regions with high occlusal load (mechanical stability priority)	Divergent neck
Immediate loading (need for high primary stability)	Microthreaded collar
Patients requiring long-term soft-tissue health and plaque control (high peri-implantitis risk / limited hygiene)	Machined collar
Limited bone width or sites with morphological constraints	Platform switching

## Data Availability

Not applicable.

## Abbreviations

MBL: Marginal bone loss
FEA: Finite element analysis
PPD: Periodontal probing depth
RCT: Randomized controlled trial
IAJ: Implant–abutment junction
